# Genome-Wide Identification of Powdery Mildew Responsive Long Non-Coding RNAs in *Cucurbita pepo*

**DOI:** 10.3389/fgene.2022.933022

**Published:** 2022-07-01

**Authors:** Jiaxing Tian, Guoyu Zhang, Fan Zhang, Jian Ma, Changlong Wen, Haizhen Li

**Affiliations:** ^1^ Beijing Vegetable Research Center (BVRC), Beijing Academy of Agriculture and Forestry Sciences (BAAFS), Beijing, China; ^2^ Key Laboratory of Biology and Genetic Improvement of Horticultural Crops (North China), Ministry of Agriculture, Beijing, China

**Keywords:** *Cucurbita pepo*, long non-coding RNA, powdery mildew, target gene, microRNA

## Abstract

*Cucurbita pepo* L. is an essential economic vegetable crop worldwide, and its production is severely affected by powdery mildew (PM). However, our understanding of the molecular mechanism of PM resistance in *C. pepo* is very limited. Long non-coding RNAs (lncRNAs) play an important role in regulating plant responses to biotic stress. Here, we systematically identified 2,363 reliably expressed lncRNAs from the leaves of PM-susceptible (PS) and PM-resistant (PR) *C. pepo*. The *C. pepo* lncRNAs are shorter in length and expressed at a lower level than the protein-coding transcripts. Among the 2,363 lncRNAs, a total of 113 and 146 PM-responsive lncRNAs were identified in PS and PR, respectively. Six PM-responsive lncRNAs were predicted as potential precursors of microRNAs (miRNAs). In addition, 58 PM-responsive lncRNAs were predicted as targets of miRNAs and one PM-responsive lncRNA was predicted as an endogenous target mimic (eTM). Furthermore, a total of 5,200 potential cis target genes and 5,625 potential trans target genes were predicted for PM-responsive lncRNAs. Functional enrichment analysis showed that these potential target genes are involved in different biological processes, such as the plant-pathogen interaction pathway, MAPK signaling pathway, and plant hormone signal transduction pathway. Taken together, this study provides a comprehensive view of *C. pepo* lncRNAs and explores the putative functions of PM-responsive lncRNAs, thus laying the foundation for further study of the regulatory mechanisms of lncRNAs responding to PM.

## Introduction

Previous transcriptome analyses of many different organisms showed that most transcribed RNAs do not have the ability to encode proteins. Based on transcript lengths, these non-coding RNAs (ncRNAs) can be mainly divided into small non-coding RNAs (sRNAs) and long non-coding RNAs (lncRNAs). LncRNAs refer to ncRNAs that are larger than 200 bp in length but have no apparent coding potential (Kapranov et al., 2007). Depending on the genomic origins relative to the genomic protein-encoding genes, lncRNAs are classified into different categories: intergenic, intronic, antisense, and sense (Ponting et al., 2009). Characteristic analysis showed that the expression level of lncRNAs was lower than that of mRNAs. In addition, there is often low evolutionary conservation and tissue-specific expression type for lncRNAs (Liu et al., 2015). Previous analyses have indicated that lncRNAs can interact with other molecules, functioning at various levels such as the transcriptional level and translational regulation (Wang and Chekanova, 2017; Lucero et al., 2021). They can regulate protein modification, chromatin remodeling, RNA metabolism, and protein modification *in vivo* through cis- or trans-activation. With the advantage of sequencing technologies, an increasing number of lncRNAs have been recognized in plants, including *Arabidopsis thaliana* (Liu et al., 2012), maize (Li et al., 2014), *Solanum lycopersicum* (Zhu et al., 2015), *Populus tomentosa* (Tian et al., 2016) and *Cucumis melo* (Gao et al., 2020). Emerging studies have indicated the important regulatory role of plant lncRNAs in different biological processes, including photomorphogenesis (Wang et al., 2014), reproduction (Wang et al., 2017), flowering (Wu et al., 2019), development of fruit (Tang et al., 2021), and stress responses (Zhu et al., 2014; Qin et al., 2017; Zhang et al., 2018; Jiang et al., 2019).

Recent research has confirmed that plant lncRNAs play vital roles in stress responses, including pathogen infection. So far, an increasing number of lncRNAs associated with pathogen infection have been found in plants, for instance, in wheat against powdery mildew (PM) (Xin et al., 2011; Zhang H. et al., 2016), in *Arabidopsis* against *Pseudomonas syringe* (Seo et al., 2017), in tomato against *Phytophthora infestans* (Cui et al., 2017; Cui et al., 2019; Jiang et al., 2019; Cui et al., 2020; Hou et al., 2020), in melon against PM (Gao et al., 2020), and in Chinese cabbage against downy mildew (Zhang B. et al., 2021). For example, it was reported that *Arabidopsis* lncRNA ELENA1 plays an important role in enhancing resistance against *Pseudomonas syringe* (Seo et al., 2017). In addition to model plants, pathogen-responsive lncRNAs have also been found in crop plants. Recently, it was reported that the cotton lncRNA (GhlncLOX3) was reported to affect the resistance to *Verticillium dahlia* by influencing the expression of *GhLOX3* related to jasmonic acid biosynthesis (Wang et al., 2021). In tomatoes, lncRNA16397 enhanced the resistance to *Phytophthora infestans* by interacting with glutaredoxin genes (Cui et al., 2017). In addition, tomato lncRNA23468 and lncRNA39026 play important roles in response to *Phytophthora infestans* infection by functioning as endogenous target mimics (eTMs) of miRNAs (Jiang et al., 2019; Hou et al., 2020). More recently, a candidate lncRNA related to downy mildew resistance was identified in Chinese cabbage (Zhang B. et al., 2021). Among the pathogens interacting with plants, there has been considerable research on PM, and several PM-related lncRNAs have been identified. In wheat, PM-induced lncRNAs were identified and characterized using a comparative expression profile analysis of PM-susceptible and PM-resistant wheat (Xin et al., 2011). In melon, several lncRNAs significantly differentially expressed after PM infection were also identified (Gao et al., 2020; Zhou et al., 2020). Moreover, 71 lncRNAs responsive to PM were identified in *Vitis vinifera* and their potential functions related to defense response were further examined (Bhatia et al., 2021). These results suggest that lncRNAs play an essential regulatory role in plant-pathogen interactions.

*Cucurbita pepo* L. (zucchini, squash) is an essential economic vegetable crop worldwide (Hafez et al., 2018). However, *C. pepo* is very susceptible to the cucurbit PM fungus. Thus far, PM has become one of the most serious diseases affecting *C. pepo* yields in the world. PM is a common and widely distributed fungal disease that affects different kinds of plants (Kusch and Panstruga, 2017; Chen et al., 2020). In cucurbit crops, powdery mildew disease is mainly caused by *Phytophthora xanthii* (Perez-Garcia et al., 2009). It can reduce the fruit yield and quality of *C. pepo* by affecting its photosynthesis, growth, and fruit development (Barickman et al., 2017). The use of chemical and biological fungicides has become one of the main measures for agricultural PM control. However, this strategy increases the resistance of PM fungi and may negatively affect the environment and human health (Zhang S. et al., 2021). Thus, it is of great significance to study the molecular mechanism of PM resistance in the molecular breeding of resistant varieties. The mechanisms of plant defense against PM have been previously reported (Ellinger et al., 2013; Hu et al., 2019). Additionally, several PM resistance-related genes have been identified in wheat (Qie et al., 2019), cucumber (Liu et al., 2017; Xu et al., 2017; Xu et al., 2019), and melon (Romero et al., 2008). In cucumber, several *MLO-like* genes have been identified as susceptibility genes to PM (Schouten et al., 2014; Berg et al., 2015). In addition, *CmMLO2* has been identified in muskmelon as a gene related to PM pathogenesis (Cheng et al., 2013). In pumpkin (*Cucurbita moschata* Duch.), *WRKY21*, *MLO3*, and *SGT1* were identified as candidate genes conferring PM-resistance (Guo et al., 2018). Moreover, the overexpression of *CmSGT1* (Guo et al., 2019) and *CmbHLH87* (Guo et al., 2020) can increase PM resistance in tobacco. Although these studies increase our understanding of the mechanism of plant resistance to PM, the molecular mechanisms underlying the PM resistance in *C. pepo* remain largely unknown. Because PM is one of the factors that severely affect *C. pepo* production, identification of PM-responsive lncRNAs in *C. pepo* is necessary.

In our study, we first conducted systematic identification of lncRNAs in *C. pepo* leaves and then described comprehensive PM-responding lncRNA profiles. In total, we identified 2,363 lncRNAs, including 242 lncRNAs differentially expressed after PM inoculation. To explore the potential function of these PM-responsive lncRNAs, we further predicted interactions between lncRNAs and miRNAs, as well as interactions between lncRNAs and genes. Functional enrichment analysis found that the potential target genes of PM-responsive lncRNAs were significantly associated with various biological processes, such as the plant–pathogen interaction pathway, MAPK signaling pathway, and plant hormone signal transduction pathway. Thus, we selected some candidate lncRNAs with potentially important roles related to PM resistance in *C. pepo*. Our results provide a rich resource for the exploration of the functional roles of *C. pepo* lncRNAs in PM resistance and lay the foundation for the molecular breeding of varieties resistant to PM.

## Materials and Methods

### Plant Materials and Powdery Mildew Inoculation

We used *C. pepo* inbred lines “PS” and “PR,” which are susceptible and resistant to PM, respectively, as the host plant. The seeds of “PS” and “PR” were provided by the National Engineering Research Center for Vegetables (NERCV), Beijing, China. The sterilized seeds were germinated on moistened filter paper in a growth chamber (28°C in darkness). The germinated seeds were then planted in 9-cm-deep plastic pots containing sterilized soil, with one seed per pot. They were grown in a growth chamber with a photoperiod of 16/8 h (day/night) at 28°C/20°C (day/night). PM fungus (*P. xanthii*) was collected from *C. pepo* naturally infected with PM and grown at the NERCV experimental farm. The powdery mildew fungus is purified by inoculating susceptible varieties. The old conidia on the highly infected leaf surfaces were removed 24 h before inoculation so that new conidia could be produced. Then a spore suspension was prepared by soaking heavily infected leaves in tap water containing 0.01% Tween-20. PM inoculation was conducted with *C. pepo* seedlings with three true leaves by spraying with a freshly prepared spore suspension (10^6^spores/ml). The inoculated seedlings were placed in a growth chamber as described above. Leaves of both PS and PR plants were harvested at 0 (control), 12, 24, and 48 h post-inoculation (hpi) with three biological replicates for each inbred line per time point. All the harvested leaves were frozen in liquid nitrogen immediately after collection and stored at −80°C until used.

### Physiological Characteristics Measurement

Six physiological characteristics were measured using leaf samples collected from PS and PR seedlings at 0, 12, 24, and 48 hpi. Total protein levels, malondialdehyde (MDA) content, the activities of superoxide dismutase (SOD), catalase (CAT), peroxidase (POD), and phenylalanine ammonia-lyase (PAL) were measured using commercial assay kits from Nanjing Jiancheng Bioengineering Institute (Nanjing, China). Detailed procedures were carried out in accordance with the manufacturer’s instructions. All the physiological characteristics above were determined using a Tecan Infinite M1000 microplate reader.

### RNA Extraction, Library Construction, and Sequencing

High-throughput RNA-seq was conducted using leaf samples collected from the PS and PR seedlings at 0 and 24 hpi. Total RNAs were extracted from all samples using TRIzol reagent (Invitrogen, CA, United States) according to the manufacturer’s instructions. After the removal of ribosomal RNA, the generation of sequencing libraries was conducted with a NEBNextR UltraTM Directional RNA Library Prep Kit for IlluminaR (New England Biolabs, United States). Finally, 12 strand-specific libraries were constructed to generate 150 bp paired-end reads on the Illumina HiSeq 4000 platform.

### Identification of lncRNAs

By removing reads containing adapters, ploy-N, and low quality, we obtained clean data. The clean reads were mapped to the *C. pepo* reference genome (version 4.1) using HISAT2 (Kim et al., 2015). The genome of *C. pepo* was downloaded from CuGenDB (http://cucurbitgenomics.org/). Then the transcriptome was assembled using StringTie software (version 1.3.1) (Pertea et al., 2016) based on reads with no more than two mismatches. All transcripts were first aligned to housekeeping ncRNA databases to exclude tRNAs, snRNAs, and snoRNAs. Then, the gffcompare program was used for the annotation of assembled transcripts, and the remaining unannotated transcripts were used to identify putative lncRNAs. The criteria used to screen lncRNA candidates are: length >200 bp and exons ≥2.Finally, the protein-coding potential was evaluated using the Coding Potential Calculator (CPC), Coding-Non-Coding-Index (CNCI), Pfam Scan (Pfam), and Coding Potential Assessment Tool (CPAT). Only transcripts with no protein-coding potential in all the four databases were identified as candidate lncRNAs. The types of lncRNAs were classified based on their genomic locations (Roberts et al., 2011).

### Characteristic Analysis of lncRNAs

To identify known lncRNAs, lncRNA sequences were subjected to BLAST searches against other plant lncRNAs in GREENC and CANTATAdb using BLASTN (coverage >80% and E-value < 1e-10). We further calculated the repetitive element and GC content of identified lncRNAs using Repeat Masker and EMBOSS explorer’s geecee tool, respectively.

### Differential Expression Analysis of lncRNAs

To identify lncRNAs related to PM resistance in *C. pepo*, the differential expression was analyzed between 24 hpi and 0 hpi for both PS and PR plants using the DESeq R package (Love et al., 2014). The fold-change (FC) was calculated, representing the change in lncRNA expression between 24 hpi and 0 hpi. The criteria for identifying differentially expressed lncRNA (DEL) is: log2FC ≥ 1 or ≤ −1 with *p*-value < 0.05.

### Predicting Cis-Regulatory Elements of PM-Responsive lncRNAs

In order to find cis-acting motifs, the 1000 bp sequences upstream of the DELs were acquired and submitted to the “Search for Care” tool of the PlantCARE database (http://bioinformatics.psb.ugent.be/webtools/plantcare/html/).

### Prediction of Interactions Between lncRNAs and miRNAs

BLASTN was used to compare all lncRNA sequences with miRNA precursor sequences (identity >90% and E-value < 1.0E-5), so as to predict the possibility of lncRNA as a potential miRNA precursor. No mismatch was allowed in the mature sequence region of miRNA. The lncRNAs potentially targeted by miRNAs were predicted using psRNATarget (https://www.zhaolab.org/psRNATarget/) (Dai and Zhao 2011), with an expectation ≤5. The eTMs for miRNAs were predicted by combining RNAhybrid (https://bibiserv.cebitec.uni-bielefeld.de/rnahybrid/) (Rehmsmeier et al., 2004) with the rules established by Hou et al. (2020). For degradome analysis, an RNA mixture from 12 leaf samples was pooled for degradome library construction and then sequenced on an Illumina HiSeq 2500 instrument (LC Sciences, Hangzhou, China).

### Prediction and Functional Analysis of Target Genes

We predicted potential target genes for PM-responsive lncRNAs based on their regulation modes. Prediction of cis target genes was done according to the positional relationship between lncRNAs and genes. The adjacent genes within 100 kb upstream or downstream of lncRNAs were regarded as potential cis target genes (Jia et al., 2010). Trans target gene prediction was conducted based on expression correlation analysis of lncRNA and mRNA. Trans target genes were genes with a correlation absolute value greater than 0.9 and *p*-value less than 0.01. Gene ontology (GO) enrichment analysis of the target genes was implemented using the topGO R packages. For pathway enrichment analysis, KOBAS (Xie et al., 2011) software was used.

### Validation of PM-Responsive lncRNAs by qRT-PCR

Eight PM-responsive lncRNAs were selected and validated using quantitative real-time PCR (qRT-PCR). Total RNA was extracted from leaf samples using Trizol reagent (Thermo Fisher Scientific, United States). Then lnRcute lncRNA First-Strand cDNA Synthesis Kit (Tiangen, China) was used for reverse transcription. The qRT-PCR reactions were conducted with lnRcute lncRNA qPCR Detection Kit (Tiangen, China) on an ABI7500 system (Thermo Fisher Scientific, Waltham, MA, United States). All qRT-PCR amplifications were carried out in triplicate. The *C. pepo actin* gene (Cp4.1LG04g06840) was used as an internal reference gene. The 2^–ΔΔ^Ct method (Livak and Schmittgen, 2001) was used for data analysis. Primers used for qRT-PCR are listed in Supplementary Table S1.

## Results

### Powdery Mildew Inoculation Affects the Physiological Characteristics of *C. pepo*

**FIGURE 1:**
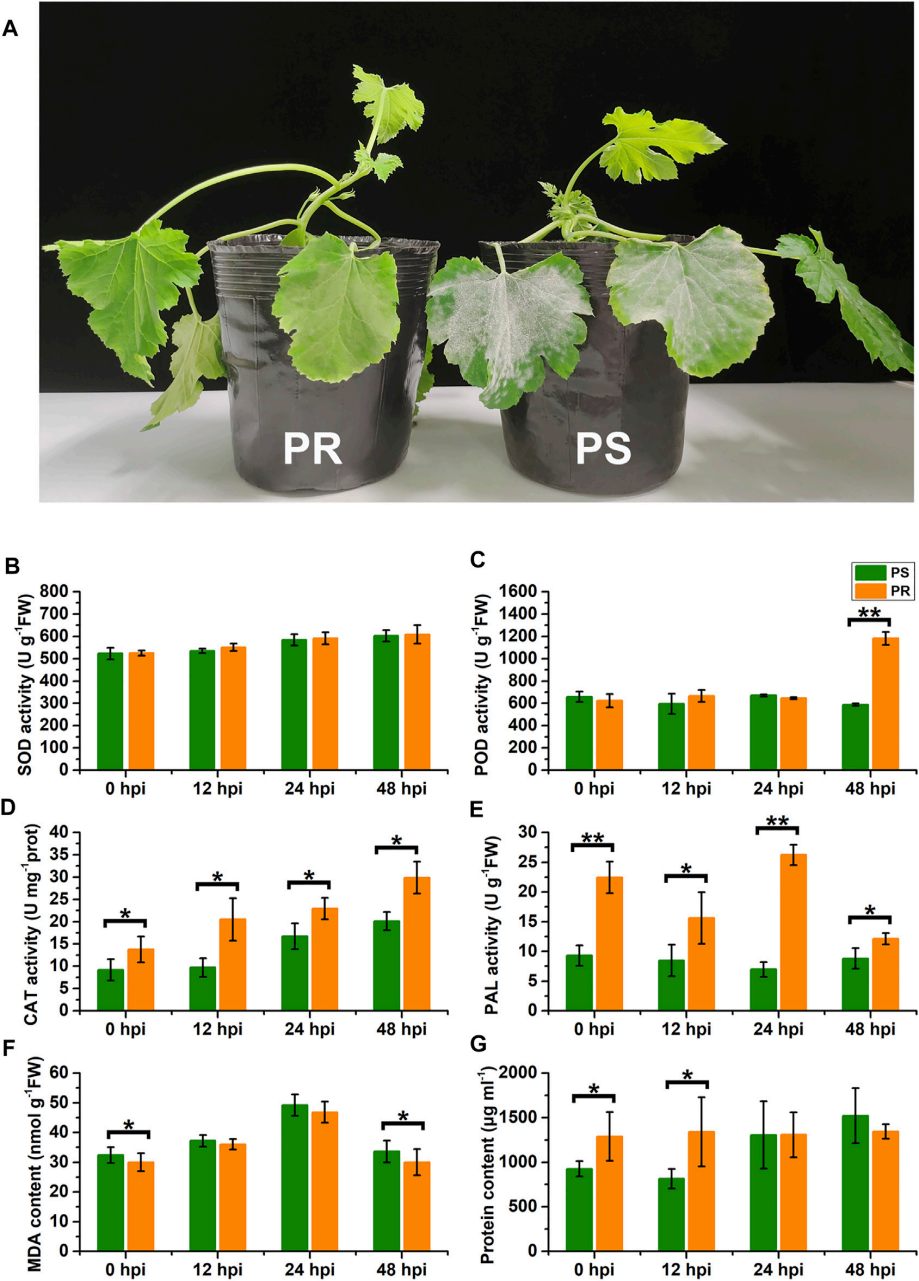
Effects of powdery mildew inoculation on physiological characteristics. **(A)** Different phenotypes of susceptible and resistant *C. pepo* at 7 days after PM inoculation. **(B–G)** Changes in superoxide dismutase (SOD) activity, peroxidase (POD) activity, catalase (CAT) activity, phenylalanine ammonia-lyase (PAL) activity, malondialdehyde (MDA) content, and total protein concentration within leaves of *C. pepo* 0, 12, 24 and 48 h post PM inoculation. PS represents PM-susceptible *C. pepo* and PR represents PM-resistant *C. pepo*. Error bars indicate standard deviations among three biological replicates (*n* = 3). Asterisks indicate significant differences between PS and PR (“*”represents *p* < 0.05; “**” represents *p* < 0.01).

To explore the influence of PM disease on the physiology of *C. pepo*, we measured the activities of SOD, POD, CAT, PAL, the MDA content, and the total protein concentration in the leaves of susceptible (PS) and resistant (PR) *C. pepo* harvested at 0, 12, 24, and 48 hpi. The phenotypes of PS and PR plants were significantly different 7 days after PM inoculation (Figure 1A). As shown in Figures 1B–G, PM inoculation affected all six physiological characteristics from 0 to 48 hpi, and some physiological characteristics significantly differed between PS and PR. For example, the POD activity of PS plants did not show a significant change after PM inoculation, but the POD activity of PR plants significantly increased at 48 hpi (Figure 1C). In addition, the CAT activity gradually increased after powdery mildew inoculation, with significant differences between PS and PR plants at 0, 12, 24, and 48 hpi, respectively. Altogether, these differences at the physiological level suggest that the transcriptome of *C. pepo* may have significantly changed after PM inoculation. Moreover, the difference between PS and PR plants in physiological characteristics suggests their different responses to PM inoculation.

### Genome-wide Identification and Characterization of lncRNAs in *C. pepo*

**FIGURE 2:**
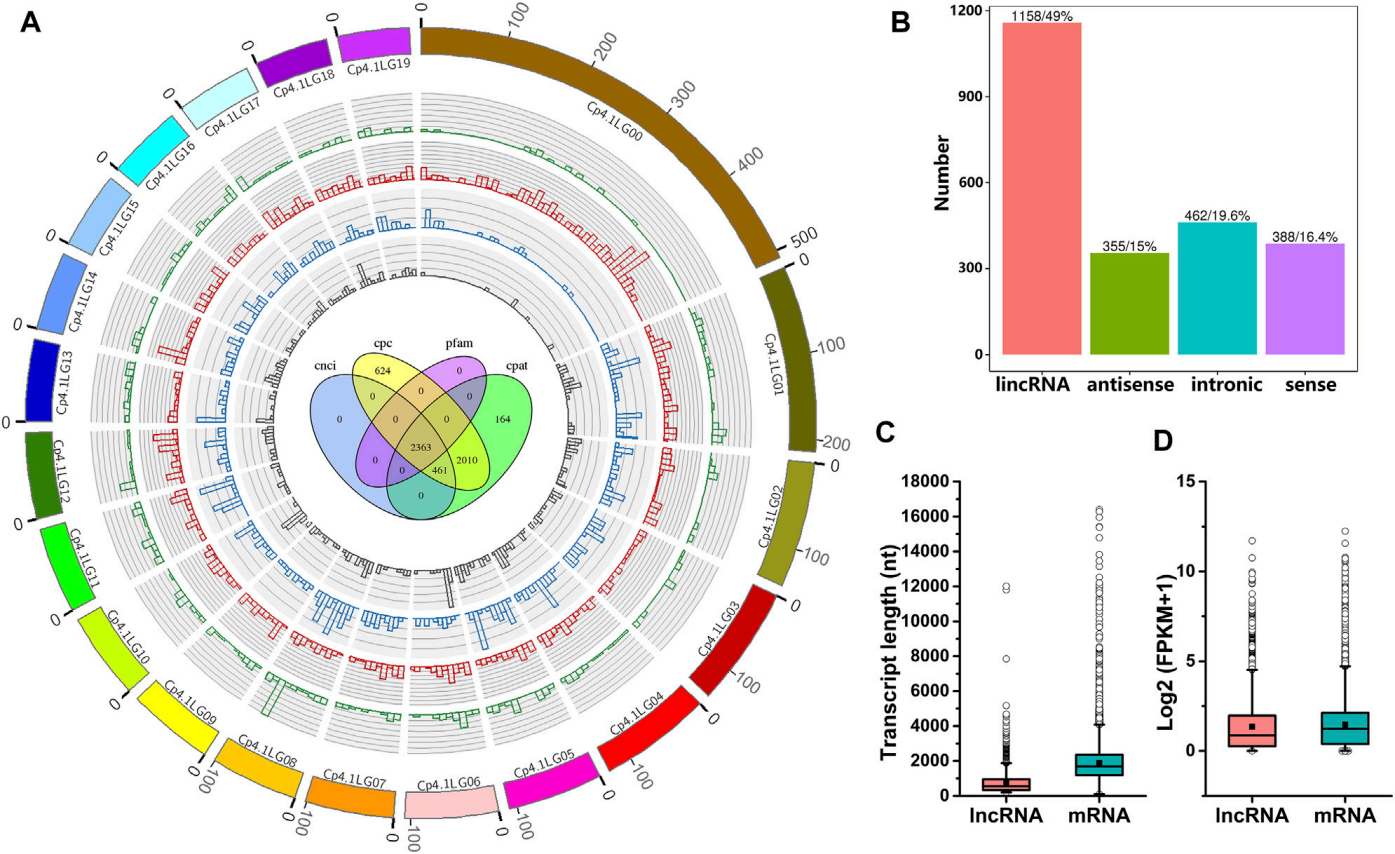
Characteristics of *C. pepo* lncRNAs. **(A)** Distribution of lncRNAs along *C*. *pepo* chromosomes. The scale unit of chromosomes is 10^5^ bp. Green represents sense lncRNAs, red represents lincRNAs, blue represents intronic lncRNAs and grey represents antisense lncRNAs. Venn diagram in the center shows the number of lncRNAs predicted by evaluating protein-coding potential in the Coding Potential Calculator (CPC), Coding-Non-Coding-Index (CNCI), Pfam Scan (Pfam), and Coding Potential Assessment Tool (CPAT). **(B)** Classification of *C. pepo* lncRNAs according to their genomic positions. **(C,D)** Comparisons between lncRNA and mRNA. lncRNAs are shorter **(C)** and expressed at lower levels **(D)** than protein-coding transcripts.

To systematically identify lncRNAs in *C. pepo*, we performed RNA sequencing using leaves of PS and PR plants at 0 and 24 hpi. Detailed information regarding RNA-Seq data is provided in Supplementary Table S2. As shown in Supplementary Table S2, approximately 93.58–95.42% of clean reads were uniquely mapped to the *C. pepo* genome. In total, 2,363 candidate lncRNAs were predicted (Figure 2A), including 1,158 lincRNAs, 355 antisense lncRNAs, 462 intronic lncRNAs, and 388 sense lncRNAs (Figure 2B). The distribution analysis showed that these 2,363 lncRNAs were evenly distributed on all the 20 *C. pepo* chromosomes (Figure 2A). The details of the 2,363 lncRNAs are provided in Supplementary Table S3.

The characteristics of *C. pepo* lncRNAs were further analyzed. BLAST analysis of *C. pepo* lncRNAs against the plant non-coding RNAs databases suggested that the overwhelming majority (99.83%) of *C. pepo* lncRNAs could be species-specific. These 2,363 lncRNAs were distributed in the *C. pepo* genome at a density of 9.73 lncRNAs per Mb. The mean GC content of the lncRNAs predicted in this study was 39.47%, and more than half of the lncRNAs (75.92%) did not contain repeated sequences. To further investigate the features of lncRNAs, the length and expression level of *C. pepo* lncRNAs were compared with mRNAs. In general, all the identified lncRNAs showed shorter lengths and lower expression levels as compared to mRNAs in *C. pepo* leaves (Figures 2C,D). For example, the mean length of *C. pepo* lncRNAs is 766 nt, which was shorter than the mRNAs of *C. pepo* (median length of 2,255 nt) (Figure 2C).

### Identification of Differentially Expressed lncRNAs After Powdery Mildew Inoculation

**FIGURE 3:**
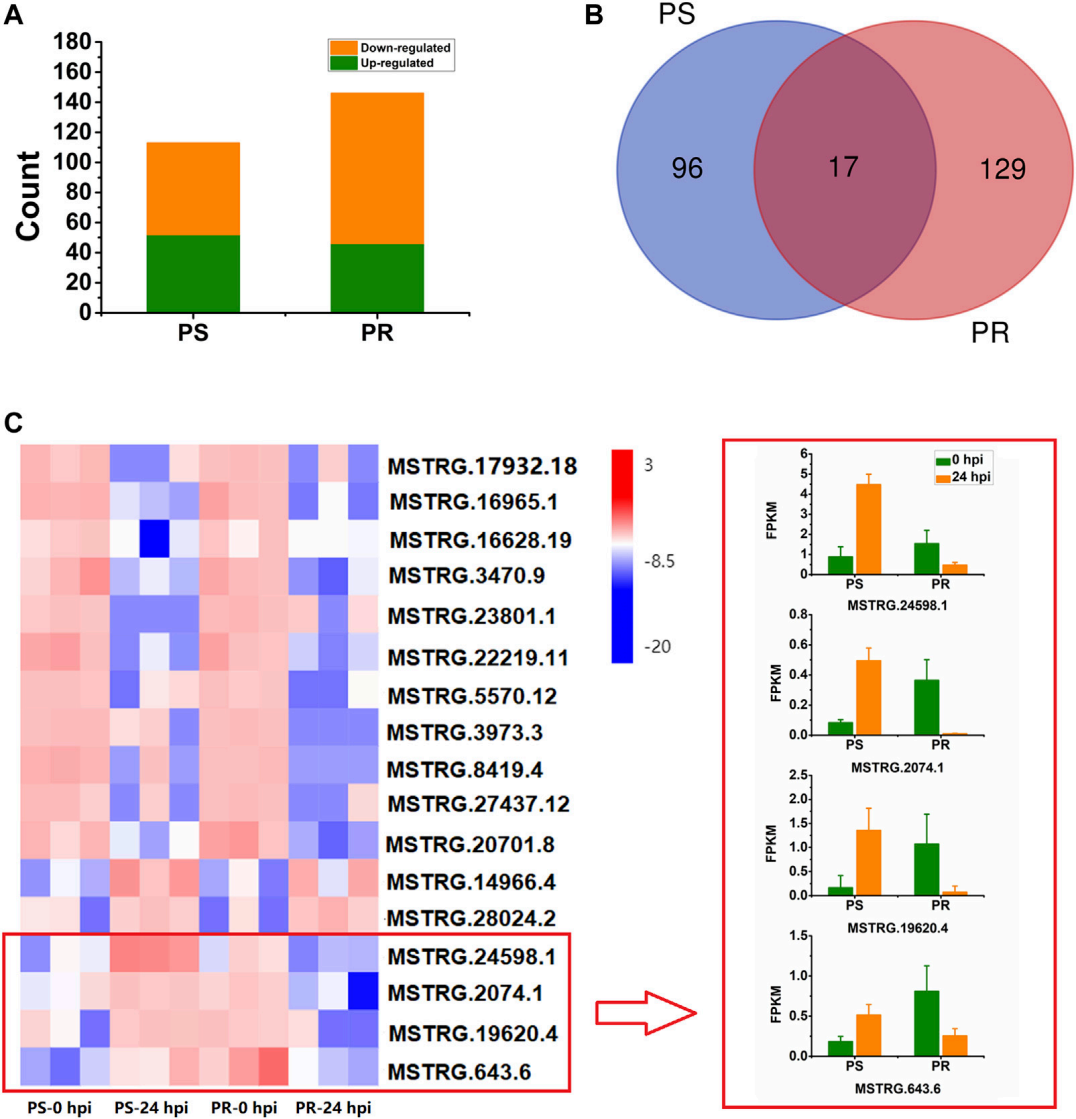
Statistical analysis and expression of PM-responsive lncRNAs in PM-susceptible and PM-resistant *C. pepo*. **(A)** Number of down- and up-regulated lncRNAs at 24 hpi in PS and PR. **(B)** Number of shared and specific PM-responsive lncRNAs in PS and PR. **(C)** Expression of the 17 PM-responsive lncRNAs shared by PS and PR. Four of them showed inconsistent expression patterns between PS and PR. PS represents PM-susceptible *C. pepo* and PR represents PM-resistant *C. pepo*. Error bars indicate standard deviations among three biological replicates (*n* = 3).

To identify lncRNAs responsive to PM infection, differential expression analysis was conducted for PS and PR plants. The number of DELs in PS at 24 hpi was 113, with 52 (46%) up-regulated and 61 (54%) down-regulated (Figure 3A, Supplementary Table S4). For PR, 146 DELs were identified at 24 hpi, with 46 (32%) up-regulated and 100 (68%) down-regulated (Figure 3A; Supplementary Table S5). Compared with PS, more DELs were identified in PR. In addition, there were more down-regulated DELs than up-regulated DELs.

In total, we identified 242 DELs after PM infection (log_2_FC > 1 or < −1, and *p* < 0.05), including 39 sense lncRNAs, 33 antisense lncRNAs, 118 lincRNAs, and 52 intronic lncRNAs. Among the 242 DELs, 96 and 129 lncRNAs were specifically differentially expressed in PS and PR plants, respectively (Figure 3B). Additionally, 17 DEL were differentially expressed both in PS and PR plants at 24 hpi (Figure 3B), with four of them regulated in different directions in PS and PR plants (Figure 3C). For example, MSTRG.24598.1 was significantly up-regulated at 24 hpi in PS plants but was significantly down-regulated in PR plants.

### Cis-Elements in the Promoters of PM-Responsive lncRNA Genes

**FIGURE 4:**
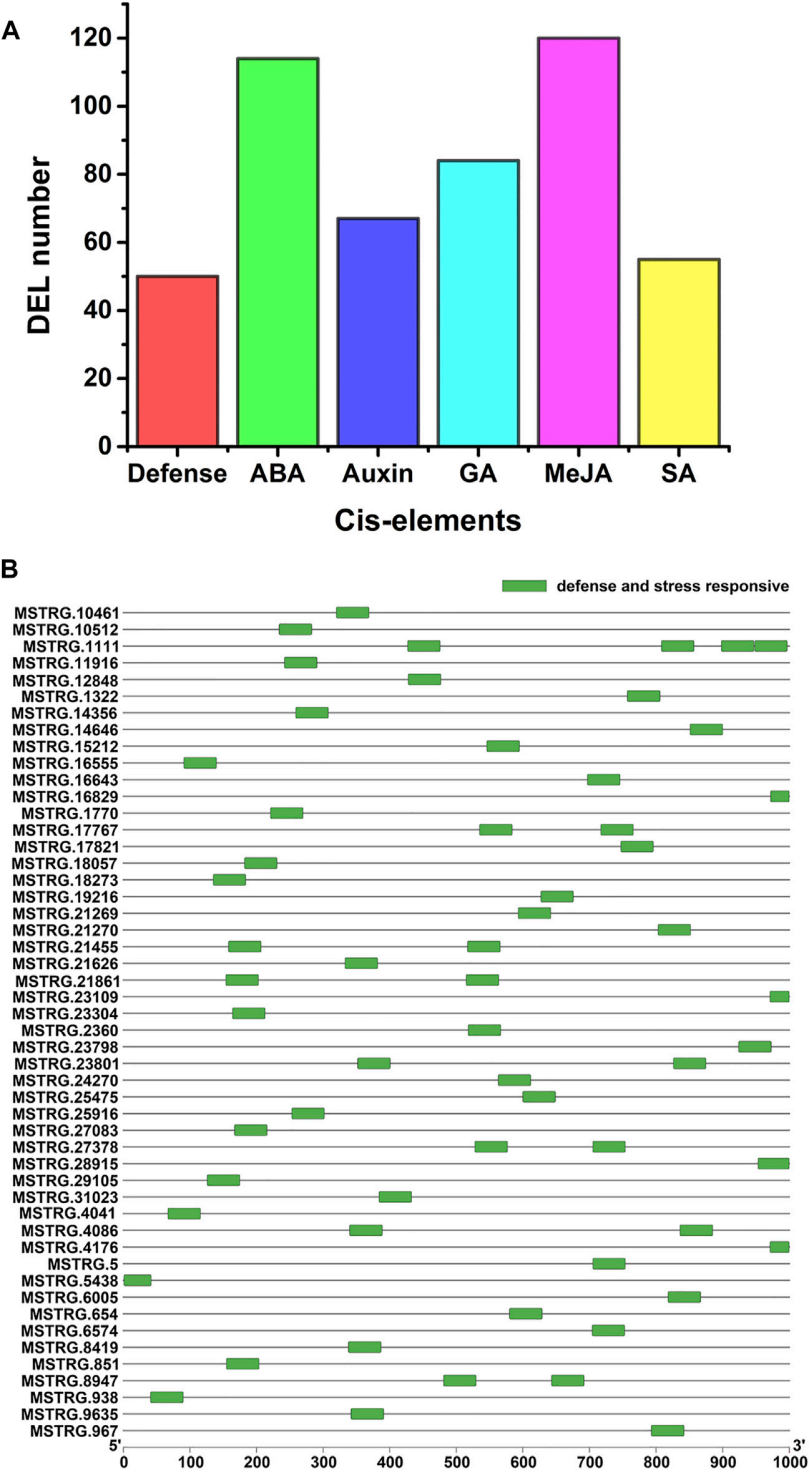
Cis-elements in the promoters of the PM-responsive lncRNA genes. **(A)** Different types of cis-acting elements were identified. Defense represents defense and stress-responsive cis-element, ABA represents abscisic acid-responsive cis-element, Auxin represents auxin-responsive cis-element, GA represents gibberellin responsive cis-element, MeJA represents Methyl Jasmonate responsive cis-element and SA represents salicylic acid-responsive cis-element. **(B)** Distribution of cis-acting elements involved in defense and stress responses in PM-responsive lncRNAs.

Cis-acting elements are related to responses to stress in plants. To identify cis-acting elements of the PM-responsive lncRNAs, we analyzed the 1000-bp upstream sequence of the 242 PM-responsive lncRNAs using PlantCARE. Some cis-acting elements associated with defense and hormone responsiveness were found in the promoter sequence of 205 PM-responsive lncRNAs (84.71%) (Figure 4A; Supplementary Table S6). As shown in Figure 4B, cis-acting elements associated with defense and stress responsiveness were identified in 50 PM-responsive lncRNAs. Among all the cis-acting elements predicted in this study (Supplementary Table S6), ABRE (abscisic acid-responsive) was the most numerous cis-acting element.

### Interactions Between lncRNAs and miRNAs

**FIGURE 5:**
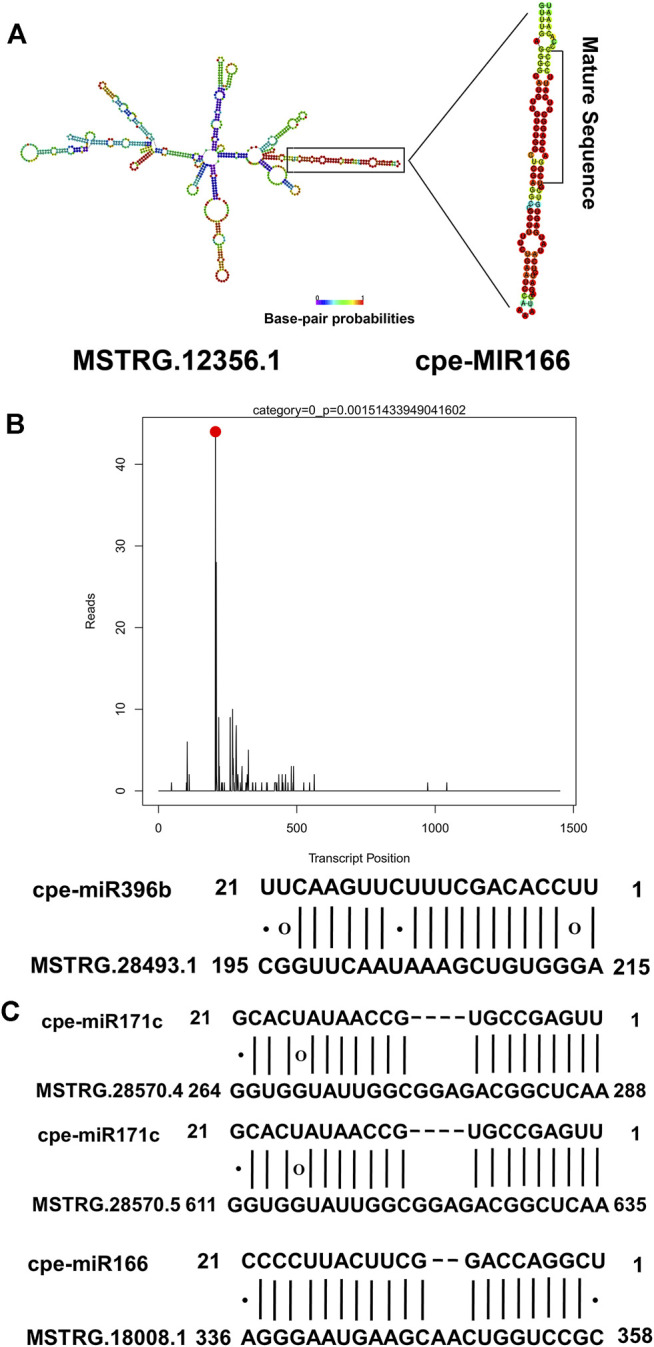
Interactions between lncRNAs and miRNAs. **(A)** MSTRG.12356.1 was predicted to be a potential precursor of cpe-miR166.**(B)** MSTRG.28493.1 was predicted as a potential target of cpe-miR396b. The degradome T-plot of MSTRG.28493.1 showed a single clear peak at the degradation site. **(C)** Three lncRNAs, including one PM-responsive lncRNA (MSTRG.28570.4) were predicted as target mimics of two miRNAs.

Because lncRNA-miRNA interactions might play a non-negligible role in resistance to disease in plants, all the lncRNAs identified in this study were used to predict potential interactions with miRNAs. In total, 34 lncRNAs were identified as potential precursors of 10 miRNAs (Supplementary Table S7), including miR166, which may play an important role in regulating plant responses to pathogens. Among the 34 lncRNAs that are potential precursors of miRNAs, six lncRNAs were PM-responsive. For example, MSTRG.12356.1, which was up-regulated in PR plants after PM inoculation, was predicted to be the precursor of cpe-miR166 (Figure 5A). A total of 312 lncRNAs were identified as potential targets of 20 miRNAs belonging to 13 families (Supplementary Table S8). Among them, eight lncRNA-miRNA interactions were verified by degradome sequencing (Supplementary Table S9). For example, MSTRG.28493.1 was targeted by cpe-miR396b. The degradome T-plot of MSTRG.28493.1 showed a single clear peak at the degradation site (Figure 5B). In the present study, it was found that three lncRNAs, including one PM-responsive lncRNA (MSTRG.28570.4), were potential target mimics of two miRNAs (Figure 5C). Our results indicated that lncRNAs may participate in the response to PM by interacting with miRNAs.

### Target Prediction of PM-Responsive lncRNAs

To analyze the potential functions of PM-responsive lncRNAs, the potential cis and trans target genes of these lncRNAs were predicted. As a result, we identified 5,200 cis target genes for 241 PM-responsive lncRNAs and 5,625 potential trans target genes for 227 PM-responsive lncRNAs (Supplementary Table S10). In addition, 30 potential target genes were predicted as both cis and trans targets of 21 PM-responsive lncRNAs. In total, 16,758 potential lncRNA-target pairs were identified. The results suggest that these PM-responsive lncRNAs in *C. pepo* may play important roles in responding to PM by interacting with their cis or trans target genes.

**FIGURE 6:**
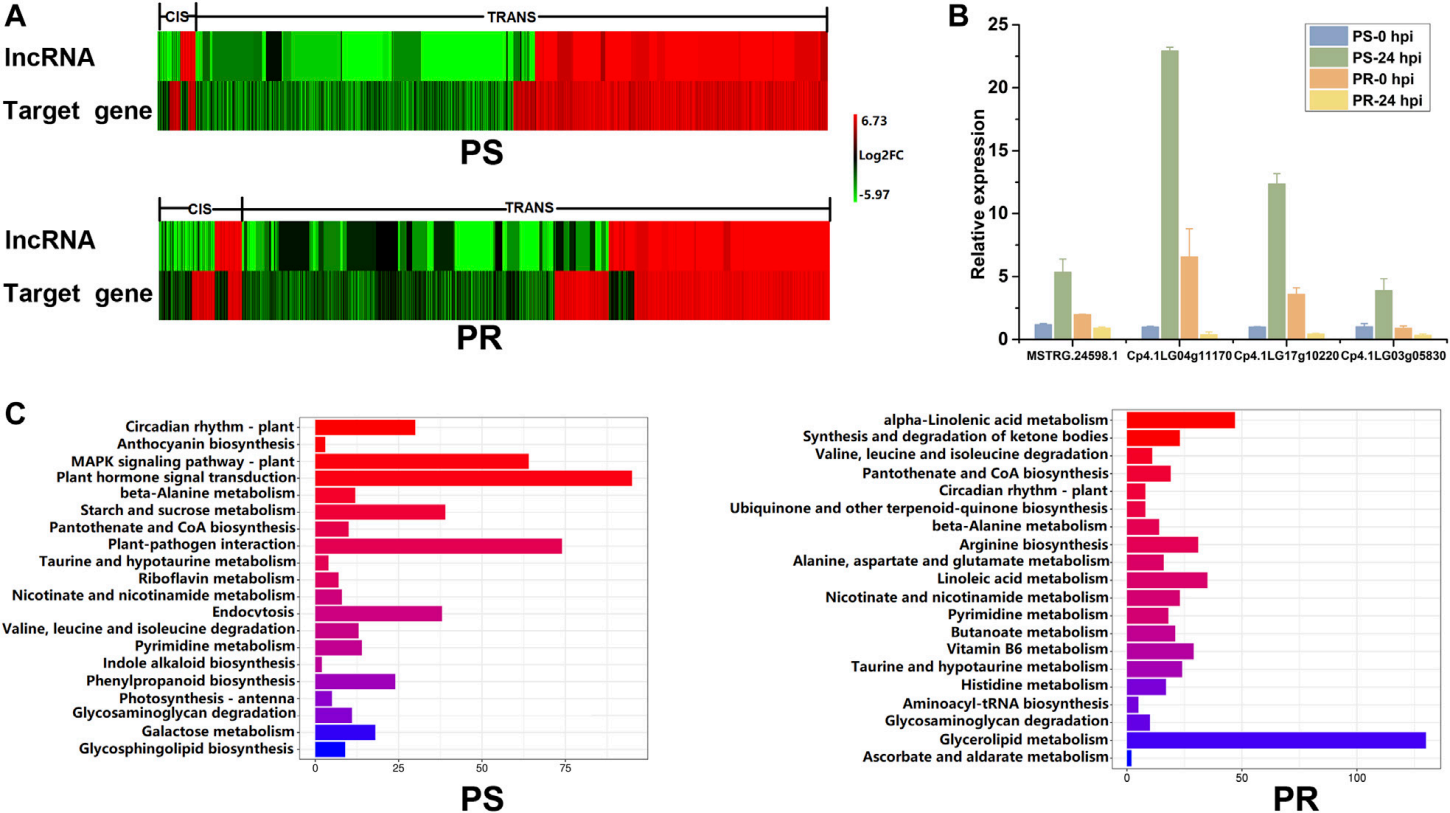
Expression and function analysis of potential target genes of PM-responsive lncRNAs. **(A)** Expression relationship between PM-responsive lncRNAs and their potential target genes. Heat map representing the log_2_FC of expression levels of PM-responsive lncRNAs and their putative target genes at 24 h post PM inoculation. **(B)** Confirmation of the expression relationship of MSTRG.24598.1 and its three putative target genes using qRT-PCR. Error bars indicate standard deviations among three biological replicates (*n* = 3). **(C)** Pathway enrichment analysis for the potential target genes of PM-responsive lncRNAs in PS and PR, respectively. The top 20 pathways were listed and shown. PS represents PM-susceptible *C. pepo* and PR represents PM-resistant *C. pepo*.

To explore the expression patterns of PM-responsive lncRNAs and their corresponding target genes, the trends of expression after PM inoculation were analyzed and compared (Figure 6A). The lncRNAs and target genes showed not only the same expression tendency but also the opposite expression tendency. The diverse expression relationships between PM-responsive lncRNAs and target genes indicate that the regulatory mechanism of lncRNAs is complex. After PM inoculation, the expression trend of most differentially expressed target genes was the same as that of the corresponding lncRNA. Among all the lncRNA-target pairs, the percentage showing the same expression trend after PM inoculation in PS and PR plants was 93.97 and 82.83%, respectively. For cis target genes, there was little difference in the number of target genes with the same or opposite expression trend as lncRNAs. However, for trans target genes, there were far more target genes with the same expression trend as lncRNAs than those with the opposite expression trend. The qRT-PCR was conducted to confirm the relationship between the expression levels of MSTRG.24598.1 and its three putative target genes (Figure 6B). As shown in Figure 6B, MSTRG.24598.1 and its target genes were up-regulated after PM inoculation in PS, but down-regulated after PM inoculation in PR. The results indicated that MSTRG.24598.1 and its targets shared the same expression trend.

### Functional Characterization of PM-Responsive lncRNAs

We next investigated the potential functions of PM-responsive lncRNAs via annotation of their potential target genes (Supplementary Table S10). Then GO and pathway enrichment analyses were conducted using the potential target genes. GO enrichment identified 196 terms and 129 terms in PS (Supplementary Table S11) and PR plants (Supplementary Table S12), respectively. Notably, some GO terms are related to response to stress, such as L-phenylalanine catabolic process, response to stress, and response to jasmonic acid. Moreover, enrichment of the “transcription factor activity, sequence-specific DNA binding” and “transcription factor TFIID complex” categories suggested that PM-responsive lncRNAs may respond to PM by interacting with transcription factors.

We further analyzed the potential target genes of PM-responsive lncRNAs using the Kyoto Encyclopedia of Genes and Genomes (KEGG) pathway analysis. The top 20 pathways are listed and shown in Figure 6C. The KEGG results showed that the target genes were mainly enriched in different metabolic pathways in PS and PR plants (Figure 6C). Among the top 20 enriched pathways, 10 pathways, including circadian rhythm-plant and plant-pathogen interaction, were significantly enriched in PS and PR plants after PM inoculation (Figure 6C). These ten pathways may play important regulatory roles in the PM response of *C. pepo*. Interestingly, the most significantly enriched pathway was circadian rhythm-plant in both PS and PR. In addition, we identified some pathways that are only enriched in PS or PR plants (Figure 6C). The pathway analysis indicated that M-responsive lncRNAs may respond to PM infection by regulating a wide range of biological processes.

### Candidate Regulation Network Involved in PM Response

**FIGURE 7:**
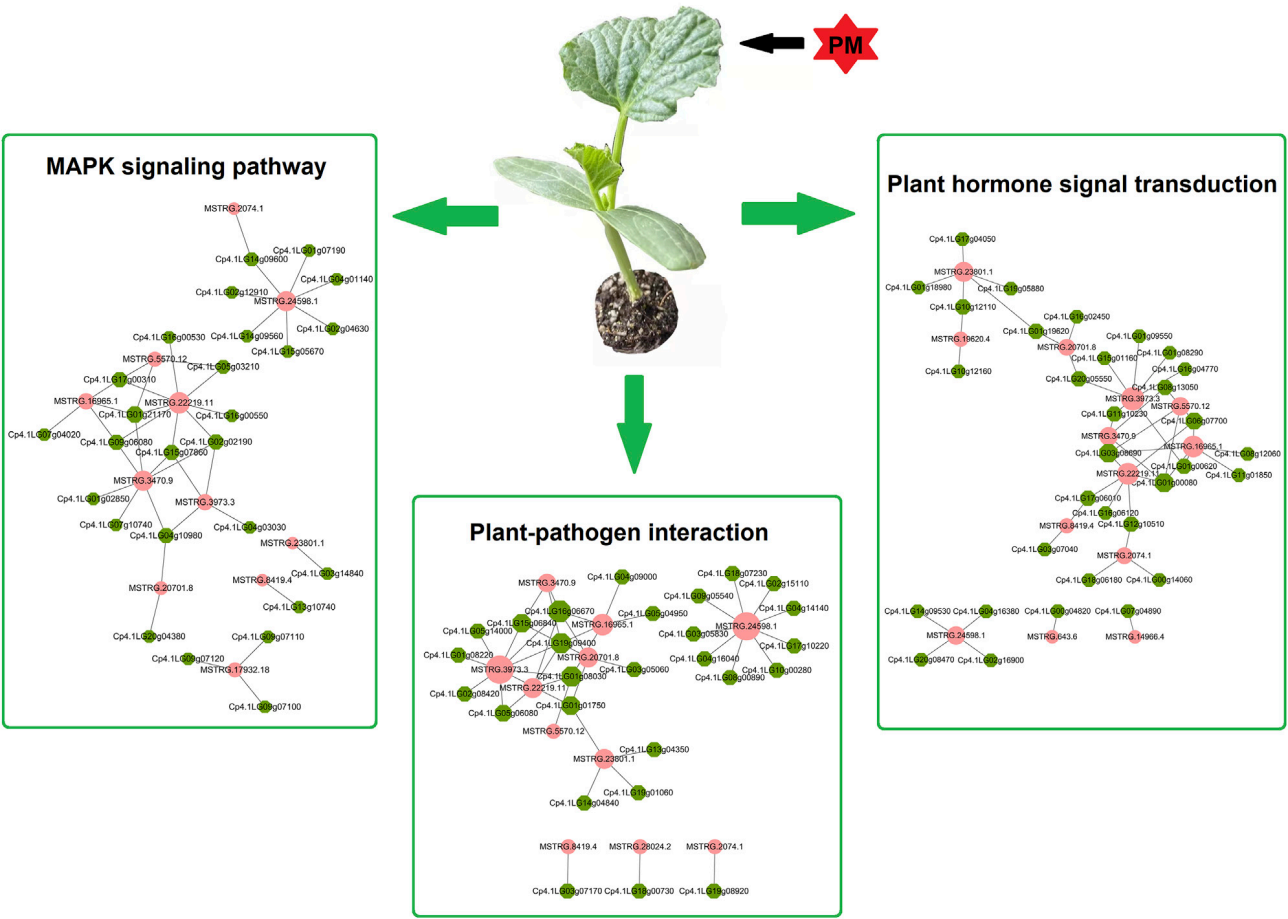
Potential regulation network of lncRNAs and target genes involved in PM response. Potential target genes of PM-responsive lncRNAs are involved in the plant–pathogen interaction, MAPK signalling, and plant hormone signal transduction pathways. The pink circles represent lncRNAs, while the green hexagons represent potential target genes.

The above functional analysis suggests that plant-pathogen interaction is one of the most important pathways in PS and PR plants. Additionally, previous studies have shown that MAPK signaling and hormone signal transduction are involved in the plant–pathogen interactions. Therefore, using PM-responsive lncRNAs shared by PS and PR, a candidate regulatory network for PM response was constructed, including the plant–pathogen interaction, MAPK signaling, and the plant hormone signal transduction pathway (Figure 7; Supplementary Table S13). This candidate regulatory network contains 131 interactions between lncRNAs and potential target genes, including 20 cis-regulated pairs, 110 trans-regulated pairs, and one both-regulated pair. In this regulatory network, only seven lncRNA-target gene pairs have a one-to-one regulatory relationship, which suggests that the regulatory relationship between lncRNA and target genes is complex. A total of 41 and 48 lncRNA-target gene pairs were involved in the MAPK signaling and plant hormone signal transduction pathways, respectively. In addition, 42 lncRNA-target gene pairs (11 lncRNAs and 27 target genes) were involved in the plant-pathogen interaction pathway. In particular, there are potential interactions for MSTRG.24598.1 with nine trans target genes, including six *calcium-dependent protein kinase* genes and three *WRKY* transcription factors.

### Validation of PM-Responsive lncRNAs

**FIGURE 8:**
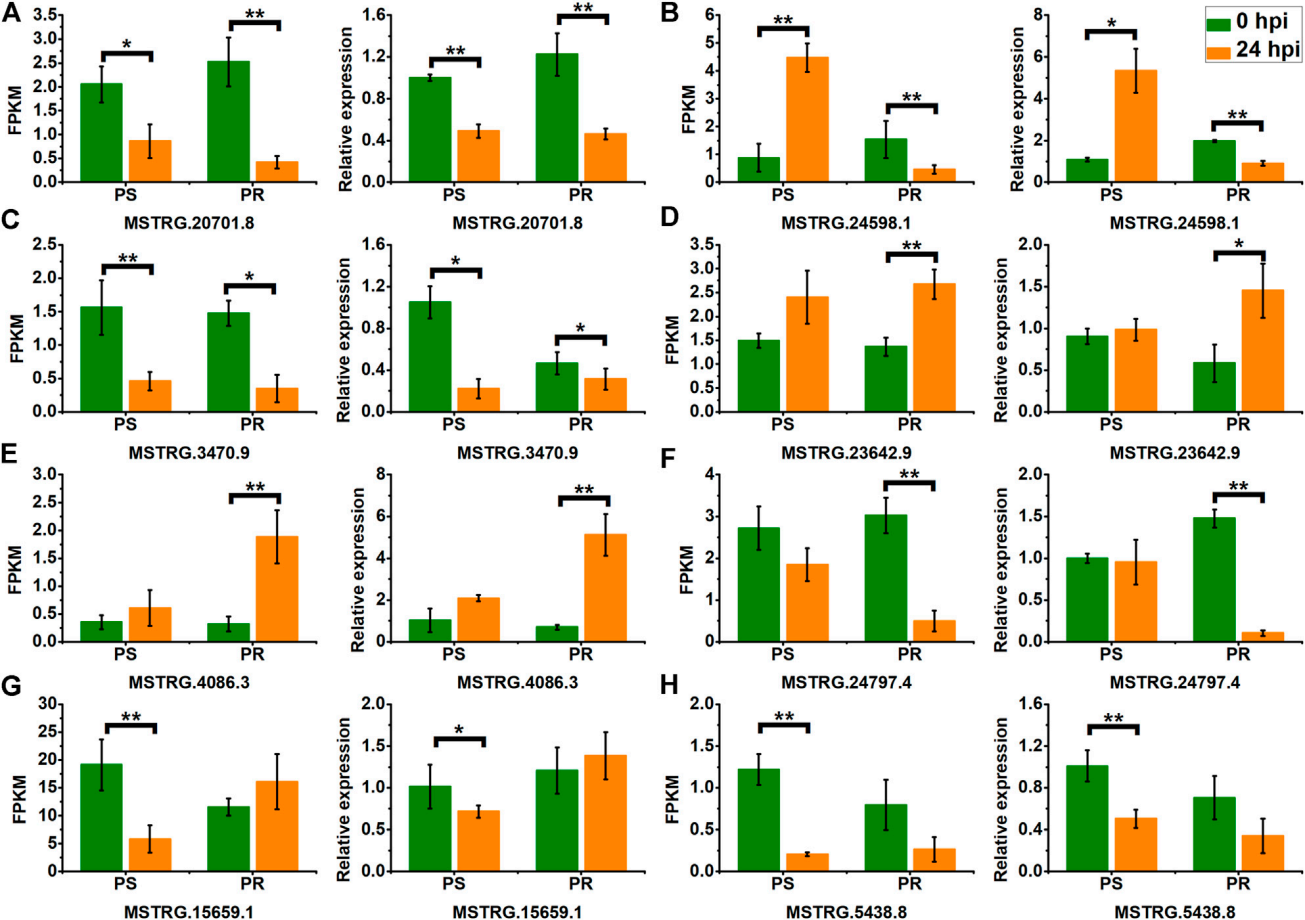
Validation of PM-responsive lncRNAs **(A–H)** using qRT-PCR. PS represents PM-susceptible *C. pepo* and PR represents PM-resistant *C. pepo*. Error bars indicate standard deviations among three biological replicates (*n* = 3). Asterisks indicate significant differences between PS and PR (“*”represents *p* < 0.05; “**” represents *p* < 0.01).

To confirm the expression pattern of PM-responsive lncRNAs, we selected eight DELs, including representatives of the four categories of lncRNAs, and conducted validation using qRT-PCR. As shown in Figure 8, the qRT-PCR results and the RNA-seq data showed a similar expression pattern for PS and PR plants, despite some differences in their expression levels. For example, the RNA-seq and qRT-PCR results showed that the expression of MSTRG.20701.8 was significantly down-regulated at 24 hpi in PS and PR plants after PM inoculation (Figure 8A). In addition, the qRT-PCR results also validated the inconsistent change patterns of MSTRG.24598.1 in PS and PR plants, consistent with the RNA-seq results (Figure 8B). These results suggest the reliability of the changing trend of lncRNA expression calculated from the RNA-seq data.

## Discussion

### Characterization of *C. pepo* lncRNAs

Transcriptome analysis has led to the discovery of non-coding transcripts that were once considered transcriptional noise. Identification of lncRNAs assists in the investigation of the gene regulatory pathways in plants. The existence of lncRNA has been reported in other plants (Liu et al., 2012; Zhang et al., 2014; Tian et al., 2016; Deng et al., 2018), but systematic identification and characterization of lncRNAs in *Cucurbita* still remains to be conducted. The recent availability of *C. pepo* genome sequences has laid a foundation for the genome-wide identification and functional analysis of *C. pepo* lncRNAs (Montero-Pau et al., 2018).

Characteristic analysis showed that the lncRNAs in *C. pepo* shared similar characteristics with the lncRNAs of other species. Previous studies have also revealed that plant lncRNAs are relatively shorter in length and have lower expression as compared to mRNAs. For example, characteristic analysis of rice lncRNAs showed that their length is far below that of mRNAs and they have relatively lower expression levels (Yu et al., 2020). In melon, it was also found that lncRNAs were shorter in length compared to protein-coding transcripts (Gao et al., 2020). Our results showed that the average length of *C. pepo* lncRNAs was only two-fifths of that of *C. pepo* mRNAs, and their expression level was much lower than that of mRNAs in *C. pepo* (Figure 2C), which is consistent with previous studies. Moreover, it has been reported that the conservation of plant lncRNAs is extremely low. For example, the majority of lncRNAs in *Capsicum annuum* were found to be novel, because these lncRNAs did not show any similarity with other lncRNAs in public databases (Baruah et al., 2021). BLAST analysis found that the vast majority (99.83%) of lncRNAs identified in this study could be species-specific. Low conservation between species has also been found in other plants (Xin et al., 2011; Liu et al., 2012; Zhang et al., 2014; Li et al., 2014), such as *Arabidopsis*, rice, maize, and wheat. The low conservation of *C. pepo* lncRNAs suggests that they may undergo rapid evolution in a manner similar to that of other plant lncRNAs. Additionally, previous studies in human, animal, and several plant species have reported that lncRNAs can interact with genes and miRNAs (Seo et al., 2017; Cui et al., 2019; Lucero et al., 2021; Tang et al., 2021). In the current study, it was predicted that *C. pepo* lncRNAs would interact with mRNAs and miRNAs. In particular, eight lnRNA-miRNA interactions were verified by degradome sequencing (Supplementary Table S9). These results indicate the existence of crosstalk between miRNAs, lncRNAs, and mRNAs in *C. pepo*. Total lncRNAs of *C. pepo* distributed throughout 20 chromosomes (Figure 2A) suggest that lncRNA is the important functional component of the *C. pepo* genome. Altogether, our results provide a comprehensive picture of lncRNAs in *C. pepo*.

### Potential Function Roles for PM-Responsive lncRNAs in *C. pepo*

So far, many studies have shown that lncRNAs can participate in plant resistance to a variety of diseases (Zhang et al., 2013; Zhang H. et al., 2016; Zhang et al., 2018; Jiang et al., 2020). PM is a severe disease of C. *pepo*, and can cause considerable loss in the yield of C. *pepo*. However, the regulatory mechanism of lncRNA in the response of C. *pepo* to PM remains to be explored. In this study, a large number of significantly differentially expressed lncRNAs were identified after PM infection, suggesting that these PM-responsive lncRNAs may assume an important role in the response of C. *pepo* to PM. Previous studies have proved that lncRNAs can play corresponding functions by interacting with target genes or miRNAs (Kung et al., 2013; Wu et al., 2013; Tang et al., 2021). Thus, we can explore the functions of candidate lncRNAs by analyzing the function of their corresponding target genes or miRNAs.

For the functional characterization of potential target genes, plant-pathogen interaction pathway highlights were obtained through pathway analysis (Figure 6C). Previous transcriptome analysis in apple and wheat indicated that the plant-pathogen interaction pathway was involved in the response to PM (Tian et al., 2019). Here, our results showed that the plant-pathogen interaction pathway was enriched in PS and PR, indicating that PM-responsive lncRNAs in *C. pepo* may participate in response to PM via regulating the expression of genes related to plant-pathogen interaction (Figure 7). In particular, it was predicted that MSTRG.24598.1 interacts with nine trans target genes related to the plant-pathogen interaction pathway. These target genes included six c*alcium-dependent protein kinase* genes and three *WRKY* transcription factors, which play an important regulatory role in responding to pathogen invasion (Lecourieux et al., 2006; Li et al., 2019; Pandey and Somssich, 2009; Wei et al., 2018; Chen et al., 2021). Moreover, several target genes of PM-responsive lncRNAs are involved in plant hormone signal transduction. For example, MSTRG.24598.1 was predicted to interact with four target genes (one cis-regulated and three trans-regulated) involved in the plant hormone signal transduction pathway. Previous studies in cucumber suggested that plant hormone signal transduction is involved in the complex regulatory network for PM resistance (Zhang P. et al., 2021; Zheng et al., 2021). These PM-responsive lncRNAs associated with plant-pathogen interaction and plant hormone signal transduction pathways are important candidates for further functional analysis of the regulatory roles of lncRNAs in the *C. pepo* response to PM.

We further investigated the potential interactions between lncRNAs and miRNAs to explore the regulatory roles of PM-responsive lncRNAs in *C. pepo*. A previous study has reported that miR166 was involved in the response to *Verticillium dahlia* in cotton (Zhang T. et al., 2016). In addition, miR166 also acts as an important regulatory factor in the response to *Tomato leaf* curl *New Delhi virus* in tomatoes (Prasad et al., 2022). Here, it was predicted that MSTRG.12356.1 which was significantly up-regulated after PM inoculation was the precursor of cpe-miR166 (Figure 5A). This indicated that MSTRG.12356.1 may participate in the response to PM by generating cpe-miR166. Three PM-responsive lncRNAs predicted to be targets of miRNAs were verified by degradome sequencing (Supplementary Table S9). Among these, MSTRG.11047.1 and MSTRG.11047.13 were potential targets of cpe-miR396a. In *Arabidopsis*, it was proven that miR396 is involved in immune responses against fungal pathogens (Soto-Suárez et al., 2017). In addition, miR396 in rice negatively regulates the resistance to rice blast disease (Chandran et al., 2019). Therefore, it indicated that MSTRG.11047.1 and MSTRG.11047.13 might respond to PM by interacting with miRNAs related to disease resistance.

### Comparison of PM-Responsive lncRNAs Between Susceptible and Resistant *C. pepo*

Considering that the effects caused by PM disease on *C. pepo* vary substantially between germplasms, a highly susceptible *C. pepo* inbred line (PS) and a highly resistant *C. pepo* inbred line (PR) to PM were selected and compared in our study. The severity of PM disease in PS and PR plants was significantly different after PM inoculation (Figure 1A), suggesting there was a significant difference in the PM resistance of PS and PR plants. Moreover, the physiological characteristics differed significantly between PS and PR plants after PM infection (Figure 1B). Altogether, the differences between PS and PR plants in phenotype and physiological characteristics suggested their different transcriptional responses to PM.

As we expected, additional PM-responsive lncRNAs were found in PR plants when compared to PS. Among the PM-responsive lncRNAs identified in PS and PR plants, only a small fraction (17 DELs) was shared by PS and PR plants (Figure 3B), which is consistent with our previous speculation that there may be significant differences in the transcriptional response between PS and PR. Interestingly, four of the 17 DELs showed inconsistent expression patterns in PS and PR plants (Figure 3C), suggesting that they may play an important role in regulating the response of *C. pepo* to PM. All four DELs were significantly up-regulated at 24 hpi in PS but significantly down-regulated at PR. In particular, it was predicted that MSTRG.24598.1 would interact with 241 potential target genes, including hormone-responsive, calcium-binding protein, and transcription factor genes. Transcription factors are important regulators in various plant biological processes, such as stress response (Jiang et al., 2017; Baillo et al., 2019). To date, increasing studies have reported the importance of *WRKY* family transcription factors in plant immunity (Pandey and Somssich, 2009), including response to PM infection (Jiao et al., 2018; Chen et al., 2021). For example, the overexpression of the transcription factor *FvWRKY42* of strawberries can enhance the resistance to PM in *Arabidopsis* (Wei et al., 2018). In the current study, the similar expression patterns of MSTRG.24598.1 and Cp4.1LG17g10220 (*CpWRKY53*) after PM infection (Figure 6B) were verified by qRT-PCR results, suggesting that MSTRG.24598.1 might participate the response to PM infection by affecting the expression of *CpWRKY53*. In addition, another trans target gene (Cp4.1LG03g05830) of MSTRG.24598.1 encoding a calcium-binding protein also showed inconsistent expression patterns in PS and PR plants (Figure 6B). In plants, calcium-binding protein participated in responses against pathogens, including fungi (Aldon et al., 2018; Li et al., 2019). Here, our results indicated that MSTRG.24598.1 may act as an important regulator in the PM resistance of *C. pepo*.

Several lncRNAs exhibit the same change trend in PS and PR, but their expression levels were different between PS and PR. For example, one PM-responsive lncRNA (MSTRG.20701.8) was down-regulated in PS and PR plants (Figure 8A). The fold changes in PS and PR plants were 0.42 and 0.17, respectively. Through target prediction analysis, MSTRG.20701.8 was co-expressed with a *Mildew Resistance Locus O* (*MLO*) gene (Cp4.1LG19g08380, trans-regulated). Consistently, the expression of Cp4.1LG19g08380 was down-regulated in PS and PR plants after PM infection, with a fold change of 0.37 and 0.14, respectively. As a result, the expression level of MSTRG.20701.8 and Cp4.1LG19g08380 in PR plants was significantly lower than that in PS plants after PM infection. It has been demonstrated that induced loss-of-function in specific *MLO* genes was associated with resistance against PM (Zheng et al., 2016; Tapia et al., 2021). For example, tomatoes can obtain high resistance against PM by inducing loss of function of *SlMLO1* (Zheng et al., 2016). These results indicated that *MLO* act as a negative regulator in the response to PM in plants. Thus, MSTRG.20701.8 might increase the PM resistance in PR plants by interacting with the *MLO* gene and decreasing its expression level.

We also identified a large number of PR-specific DELs, which might also play a critical role in resisting PM. Besides PM-responsive lncRNAs shared by PS and PR, a further functional study will be focused on these PR-specific DELs that may contribute to the resistance phenotype of PR. Altogether, our findings will contribute to further understanding of the regulatory mechanisms of lncRNAs in the response of *C. pepo* to PM. These results indicate that there might be critical regulatory functions for lncRNAs in PM resistance by positively and negatively interacting with their corresponding target genes. Subsequently, further molecular studies are required to explore the regulatory mechanisms of these candidate PM-responsive lncRNAs in resisting PM infection.

## Data Availability

The raw sequence data have been uploaded to the Genome Sequence Archive (GSA) in the National Genomics Data Center, Beijing Institute of Genomics, Chinese Academy of Sciences (https://bigd.big.ac.cn/), under accession number CRA006731.

## Supplementary Material

Supplementary Table 1 Primers used for real-time quantitative PCR.

Supplementary Table 2 Summary of C. pepo RNA-seq data.

Supplementary Table 3 Characteristics of all lncRNA identified in this study.

Supplementary Table 4 PM-responsive lncRNAs in PS.

Supplementary Table 5 PM-responsive lncRNAs in PR.

Supplementary Table 6 Cis-elements in the promoters of the PM-responsive lncRNAs.

Supplementary Table 7 lncRNAs corresponding to miRNA precursors.

Supplementary Table 8 The lncRNAs predicted as targets of miRNAs.

Supplementary Table 9 Targets of miRNAs verified by degradome sequencing.

Supplementary Table 10 The potential target genes of PM-responsive lncRNAs.

Supplementary Table 11 Enriched GO terms in PS.

Supplementary Table 12 Enriched GO terms in PR.

Supplementary Table 13 Candidate regulation network involved in PM response.
